# A reconfigurable all-optical ultrasound transducer array for 3D endoscopic imaging

**DOI:** 10.1038/s41598-017-01375-2

**Published:** 2017-04-26

**Authors:** Erwin J. Alles, Nora Fook Sheung, Sacha Noimark, Edward Z. Zhang, Paul C. Beard, Adrien E. Desjardins

**Affiliations:** 1 Department of Medical Physics and Biomedical Engineering, University College London, London, WC1E 6BT UK; 2 Materials Chemistry Research Centre, UCL Department of Chemistry, London, WC1H 0AJ UK

## Abstract

A miniature all-optical ultrasound imaging system is presented that generates three-dimensional images using a stationary, real acoustic source aperture. Discrete acoustic sources were sequentially addressed by scanning a focussed optical beam across the proximal end of a coherent fibre bundle; high-frequency ultrasound (156% fractional bandwidth centred around 13.5 MHz) was generated photoacoustically in the corresponding regions of an optically absorbing coating deposited at the distal end. Paired with a single fibre-optic ultrasound detector, the imaging probe (3.5 mm outer diameter) achieved high on-axis resolutions of 97 μm, 179 μm and 110 μm in the *x*, *y* and *z* directions, respectively. Furthermore, the optical scan pattern, and thus the acoustic source array geometry, was readily reconfigured. Implementing four different array geometries revealed a strong dependency of the image quality on the source location pattern. Thus, by employing optical technology, a miniature ultrasound probe was fabricated that allows for arbitrary source array geometries, which is suitable for three-dimensional endoscopic and laparoscopic imaging, as was demonstrated on *ex vivo* porcine cardiac tissue.

## Introduction

Biomedical ultrasound imaging has conventionally been performed with one- or two-dimensional arrays of electronic transducer elements to transmit and receive acoustic waves. These arrays, which typically comprise piezoelectric^1, 2^ or capacitive micromachined ultrasound transducers (CMUTs)^3^, have fixed geometries and acoustic properties. Significant limitations arise from this approach; one pertains to individual elements: their centre frequencies and bandwidths are determined by their size and shape, since transmission and reception typically derive from mechanical resonance. As such, an element is limited to operate either at high frequencies for high resolution and low imaging depth, or at low frequencies for low resolution and high imaging depth. A second limitation stems from the periodicity in the arrangements of the elements. To increase sensitivity and facilitate fabrication, elements larger than half the acoustic wavelength are typically used, resulting in grating and side-lobes in ultrasound images that degrade contrast^4^. Moreover, the electronic connections and associated electronic circuitry required for each element typically introduce significant complexity and cost to the manufacturing process^2^. Due to miniaturisation requirements and occasional cost sensitivities associated with single-use medical devices, these limitations are particularly prominent for minimally invasive ultrasound probes^5–8^.

Several studies have highlighted all-optical transducers as viable alternatives to their electronic counterparts for biomedical imaging^9–20^. In these probes, ultrasound is generated photoacoustically^21^ by illuminating an optically-absorbing material with excitation light; optical acoustic detection can be performed with photonic structures such as a Fabry-Pérot cavity^12, 13, 16, 22, 23^ or a micro-ring resonator^15, 24^ in which the optical reflectivity varies with deformations induced by impinging ultrasound waves. As photoacoustic ultrasound generation is non-resonant, the frequency range is determined by the bandwidth of the optical excitation modulation; consequently very high acoustic bandwidths (>100 MHz) can be achieved^11, 25^. Alternatively, by modifying the bandwidth of the excitation light through temporal modulation, the frequency and bandwidth of the optically generated ultrasound can be optimised to achieve the desired resolution and imaging depth^26^. Additionally, optically generated acoustic pressures can be higher than those achieved with piezoelectric or CMUT technologies^20, 27^. Geometric focussing using a curved, optically absorbing surface allows for spatial confinement of the transmission beam^19^, as well as sufficiently high pressures (tens of MPa) to perform therapeutic high-intensity focussed ultrasound (HIFU)^28^. Unlike arrays of electronic transducer elements, the locations and sizes of the regions in which ultrasound is generated can be varied during operation by changing the spatial patterns of excitation light provided to the optically-absorbing material. An absence of electronic components allows for immunity to electromagnetic interference and confers MRI compatibility.

In previous studies, we demonstrated that high-resolution images of biological tissue can be obtained with fibre-optic all-optical ultrasound transducers^18–20^. In these studies, two optical fibres were used for transmission and reception, respectively. This pair was mechanically scanned to create a synthetic aperture. However, this type of transducer translation is not suitable for interventional imaging, as the required actuators are typically bulky and slow relative to physiological motion.

In this study, we developed a solid-state, all-optical ultrasound probe for three-dimensional (3D) imaging. This probe exhibited a flexible geometry, a diameter compatible with the working channel of an endoscope, and did not contain moving parts at the distal end. Excitation light was delivered through a coherent optical fibre bundle to an optically absorbing coating deposited at the distal end where ultrasound was generated photoacoustically, while ultrasound detection was performed at a single fixed location. By scanning excitation light across the proximal end of the bundle, acoustic sources could be generated in arbitrary locations, which allowed for a direct comparison of the image quality achieved for various source array geometries using the same imaging system. To demonstrate the flexibility of the presented approach, and the influence of the source array geometry on the image quality, four periodic and aperiodic sets of acoustic source array geometries commonly used in endoscopic ultrasound were implemented by varying the optical scan pattern, and the corresponding point spread functions and image quality of the system were determined using point-like and extended objects. Finally, the suitability of the probe for endoscopic imaging was demonstrated through the imaging of *ex vivo* tissue.

## Methods

The coherent fibre bundle (core diameter: 12 μm, lumen diameter: 2.70 mm, outer diameter: 3.15 mm, minimum bend radius: 450 mm; Fig-50, Fujikura, Japan) achieved a one-to-one mapping between the spatial distribution of excitation light at the proximal end and ultrasound transmissions at the distal end. Via a pair of orthogonal galvo-mirrors (GVSM002, Thorlabs, Germany), collimated excitation light (beam diameter: 2.6 mm) was focussed (focal length: 50 mm; AC254-050-A, Thorlabs, Germany) onto the proximal end of the fibre bundle. Using this scanning configuration, a light spot was projected on the optically absorbing coating, which could be arbitrarily positioned across the distal end face to scan a finite acoustical source aperture.

### Experimental setup

**Figure 1:**
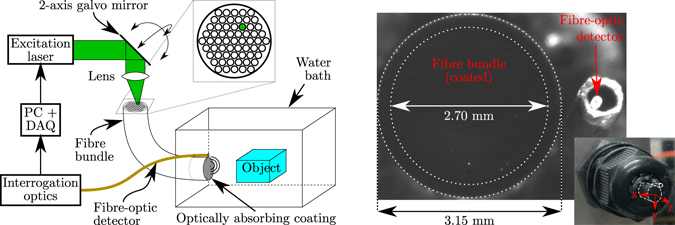
**Left:** Schematic of the experimental set-up. A two-axis galvo-mirror and lens are used to focus and arbitrarily steer laser light onto a small area of the proximal end of a coherent fibre bundle. The fibre bundle transports the focussed light to the corresponding area of an optically absorbing coating deposited at its distal end, where ultrasound is generated photoacoustically. Acoustic pulse-echo signals are recorded using a fibre-optic receiver. **Right:** Micro- and macroscopic photographs of the coated, distal end of the fibre bundle. The coordinate system used throughout this study is indicated. The ultrasound-generating area is limited by the inner dotted circle; the outer circle delineates the outer edge of the fibre bundle.

A pulsed laser (wavelength: 532 nm, pulse length: 10 ns, pulse energy: 8.6 μJ, repetition rate: 1 kHz; FQ-200-20-V-532, Elforlight, UK) was used to generate ultrasound waves. An optically absorbing coating (Carbon Black Professional Spray Paint, Liquitex, OH, USA), deposited on the distal end of the fibre bundle, converted the absorbed optical energy into acoustic energy through the photoacoustic effect. Back-scattered ultrasound was recorded using a fibre-optic acoustic receiver comprising a high finesse Fabry-Pérot cavity fabricated on the distal end of an optical fibre, which exhibited a high bandwidth and low directivity^23^. This receiver was interrogated by measuring the cavity’s reflectivity using a continuous-wave tunable laser (TUNICS T100S-HP, Yenista, France) tuned to the wavelength corresponding to the peak derivative of the cavity transfer function^29^. Acoustic data were amplified (+20 dB; DHPVA-200, Femto, Germany) and subsequently sampled using a high-speed data acquisition card (250 MSa/s, 14-bit; M4i.4420-x8, Spectrum, Germany). The experimental set-up (Fig. 1) was controlled using a custom LabVIEW script (LabVIEW 2014, National Instruments, TX, USA).

Pulse-echo responses (A-scans) were recorded for each discrete source location, thus a finite acoustical source aperture was sequentially scanned. The Fabry-Pérot cavity was found to be positioned a distance of 2.152 mm away from the centre of the fibre bundle using a high-magnification camera (DCC3240N + MVLCMC/MVL20A/MVL6X12Z, Thorlabs, Germany), and the *x* and *y* coordinates were determined to within ±25 μm. Prior to reconstruction, power-law time gain compensation was applied to compensate for geometrical attenuation. Specifically, the time-gain compensated A-scan *S*_*i*_(*t*) was computed as *S*_*i*_(*t*)=*t* ^*α*^ *A*_*i*_(*t*), where *A*_*i*_(*t*) is the A-scan generated by source position *i*, *t* is time (s) and the time gain exponent *α* was determined empirically as described below. The A-scans were reconstructed into a 3D image using the delay-and-sum algorithm^30^ (which is equivalent to dynamic focussing^31^), assuming omni-directional, point-like sources and detectors and a homogeneous speed of sound. The image amplitude $I(\vec{r})$ in each image location $\vec{r}$ was given by

1 $$I(\vec{r})=\sum _{i\mathrm{=1}}^{N}{S}_{i}(t=\frac{|\vec{r}-{\vec{r}}_{s,i}|+|{\vec{r}}_{d}-\vec{r}|}{c}),$$

where ${\vec{r}}_{s,i}$ is the position of source *i*, ${\vec{r}}_{d}$ is the position of the detector, *c* = 1480 m/s is the speed of sound, and *N* is the number of acoustic sources.

### System characterisation

The acoustic performance of the ultrasound-generating coating was assessed using a calibrated needle hydrophone (75 μm, Precision Acoustics, UK). A motorised field scan (4 mm × 4 mm grid, 35 μm stepsize; MTS50/M-Z8 + TDC001, Thorlabs, Germany) centred around the fibre bundle was performed without averaging at a distance of 2 mm, and the measurement corresponding to the highest recorded amplitude was used to determine the acoustical bandwidth (defined by the −6 dB power level) of the system. Field measurements were performed for 100 different acoustic source positions, and each field measurement was numerically back-propagated^32^ to the fibre bundle surface to determine both the acoustic pressure generated at the surface and the dimensions of the acoustical sources (measured as the FWHM of the back-propagated pressure distribution).

The spatial resolution of the imaging system was determined both numerically and experimentally by measuring the spatial extents of the point spread function throughout the imaging volume. Experimentally, this was achieved by positioning the tip of a metal pushpin (tip diameter: 50 μm) at a distance of either *z* = 0.9 mm or *z* = 2.4 mm from the fibre bundle, and scanning along a grid in the *x* and *y* directions (9 × 9 pin positions, step size: 0.5 mm) while acquiring 3D images for each discrete pin position. The point spread function was recovered from the full-width-at-half-maximum (FWHM) of each image relative to the corresponding maximum amplitude of that image. In simulations, this experiment was repeated using a point scatterer modelled as a Dirac delta distribution. The forward acoustic field at the location of the point scatterer was computed using the impulse response for a disc (diameter: 125 μm) implemented in the FOCUS package^33^; the back-scattered field recorded by the point-like acoustical receiver was obtained using the free space Green’s function^34^. The measured acoustic pulse shape was incorporated by means of temporal convolution. To account for the difference in size of the scatterers, different time gain exponents were used upon reconstruction of experimental (*α* = 1.5) and numeric data (*α*= 2.0); these values were found empirically to minimise the dependence of the pulse-echo amplitude on the location of the acoustic sources.

To study the influence of the acoustic source array geometry on the image quality, numerical and experimental results were obtained for four acoustic source location patterns. The first pattern consisted of a circle following the outer edge of the acoustical aperture to mimic a geometry commonly used in forward-looking interventional ultrasound probes^35^. The second pattern comprised a rectangular grid, as this is the common geometry for piezoelectric or CMUT matrix array transducers^2, 3^. Two additional patterns were studied in an attempt to reduce the artefacts associated with incomplete or periodic aperture scanning: one pattern comprising fourteen equidistant and concentric circles that exhibited lower degrees of periodicity than the rectangular pattern, and one fully aperiodic pattern where the source locations were randomised across the acoustical aperture. The two circular and the randomised patterns comprised 573 source locations each; the rectangular pattern comprised 576 source locations. For the circular pattern this resulted in a source spacing of approximately 14.5 μm, while for the three remaining patterns the mean spacing between sources was approximately 100 μm.

### Imaging

The 3D image quality of the system was demonstrated on a phantom consisting of a looped metal wire (wire diameter: 590 μm, loop diameter: 1.5 mm) placed at a distance of approximately 1.8 mm or 3.3 mm from the fibre bundle. The same four ultrasound transmission patterns (containing either 573 or 576 source locations) as used for the spatial resolution measurements were applied, as well as the same optical excitation and acoustical detection parameters. A time gain exponent *α* = 1.4 was used during image reconstruction. Finally, a volumetric image was acquired (using 10-fold averaging) of the inner wall of *ex vivo* porcine left ventricular heart tissue submerged in water.

## Results

### Acoustical performance

**Figure 2:**
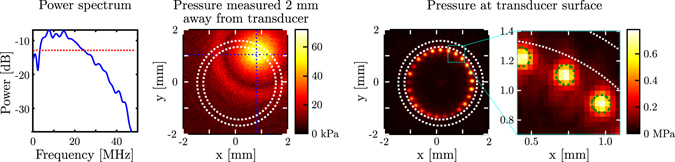
**Left:** Power spectrum (relative to 1 MPa) of the acoustic field transmitted by the optically absorbing coating, measured at a distance of 2 mm in front of the illuminated area. The dotted red line indicates the −6 dB level used to measure the bandwidth. **Middle:** Peak acoustic pressure measured across a plane placed 2 mm away from the distal fibre bundle end. The dotted blue lines indicate the location of the illuminated area on the coating; the inner and outer dotted white circles delineate the ultrasound-generating aperture and outer edge of the fiber bundle, respectively. **Right:** Compound image of the peak acoustic pressures, measured at the surface of the distal end of the fibre bundle, for 25 different acoustical source locations. The dotted green contours indicate, for each source position, the full-width-at-half-maximum (FWHM) of the acoustic pressure and are representative of the size of the acoustical sources.

Field measurements of the optically generated acoustic waves yielded a high fractional bandwidth of 156% centered around 13.5 MHz (Fig. 2, left), corresponding to a centre wavelength of 108 μm, and pressures of up to 70 kPa were recorded at a distance of 2 mm (Fig. 2, middle). Numerical back-propagation of the measured fields for 25 ultrasound source positions revealed that the pressure distribution at the fibre bundle surface was nearly circular for each source position, and that each acoustic source had an effective diameter of approximately 125 μm (Fig. 2, right). The observed spatial variations in the amplitude of the acoustic sources measured at the coated distal fibre bundle end (ranging between 0.22 and 0.78 MPa) were due to inhomogeneities in the coating thickness (cf. Supplementary Fig. 1).

### System characterisation

**Figure 3:**
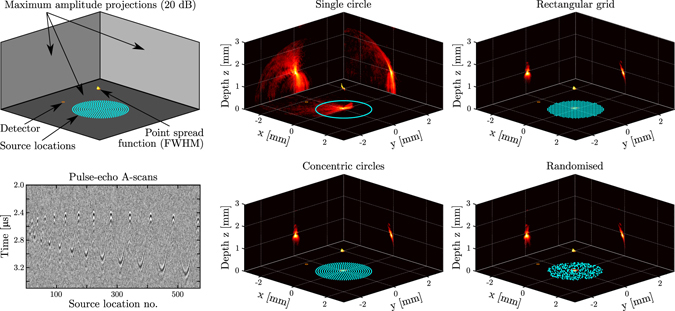
Images of the tip of a pushpin (see Fig. 4) obtained with the all-optical, three-dimensional (3D) pulse-echo ultrasound imaging system. Light was focussed sequentially into areas corresponding to each source location, and pulse-echo signals originating from each source location were recorded using an optical acoustic receiver and subsequently reconstructed into a 3D image. **Top left:** Schematic highlighting the information contained in the two rightmost columns. The source locations are indicated in light blue; the receiver in orange. The volumetric render shows the full-width-at-half-maximum (FWHM) isosurface that corresponds to the spatial point spread function. The three panels show the maximum amplitude projections along each spatial axis, displayed on a logarithmic scale with a 20 dB dynamic range. **Bottom left:** Pulse-echo A-scans recorded (using 10 averages) using a scan pattern consisting of fourteen equidistant concentric circles (573 source locations), where the circles were scanned in order of increasing radius. **Middle and right columns:** Imaging results for four different illumination patterns. In a clock-wise orientation: a single circle along the outer edge of the acoustical aperture (573 source locations), an equidistant rectangular grid covering the full acoustical aperture (576 source locations), a randomised pattern covering the full acoustical aperture (573 source locations), and a pattern comprising fourteen equidistant concentric circles (573 source locations).

Point spread functions obtained experimentally for four different source location patterns revealed the high spatial resolution obtained on-axis (*x* = *y* = 0 mm) with the system (Fig. 3). For an object depth of *z* = 0.9 mm and a source location pattern comprising concentric circles, the FWHM of the point spread function in the *x*, *y* and *z* directions measured 97 μm, 179 μm and 110 μm, respectively. Similar FWHM values were found for all four patterns. These similarities were expected, given that the point spread function is primarily determined by the acoustic bandwidth, frequency, attenuation, and aperture size, which were identical for all four patterns. However, the point spread functions evaluated at the −20 dB level varied strongly with the source location pattern. The discrepancy between the resolutions in the *x* and *y* directions were likely caused by a slight misalignment of the pushpin and surface roughness of its tip. Significantly lower artefact levels, and hence higher contrasts, were achieved with all three patterns covering the full acoustic aperture, as compared to those obtained with a circular scan pattern. When the point target was placed at a depth of *z* = 2.4 mm (Supplementary Fig. 2), slightly larger FWHM point spread function extents were obtained, while a lower signal-to-noise level was observed due to increased geometrical attenuation.

**Figure 4:**
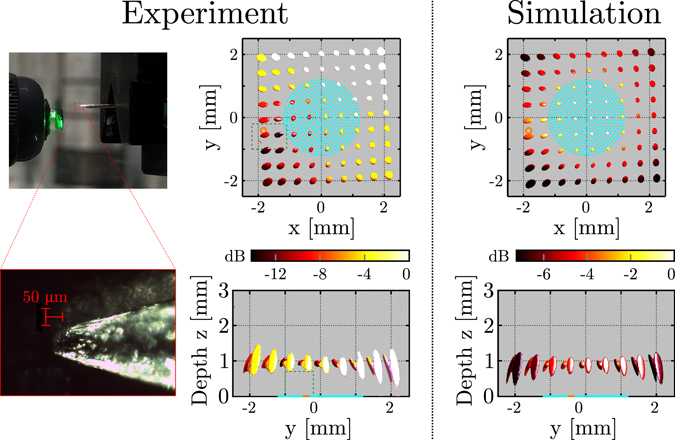
Measurement and simulation of the spatial variation of the point spread function. In the experiments, a single pushpin tip was positioned in 9 × 9 positions spaced 0.5 mm apart, and at each position a three-dimensional (3D) image of the pushpin tip was obtained. The illumination pattern (indicated in light blue) comprised 573 source locations distributed along fourteen equidistant concentric circles. The receiver position is indicated in orange. **Left column:** Macro- and microscopic photographs of the pushpin. **Middle column:** Compound image displaying the full-width-at-half-maximum (FWHM) isosurfaces of all 81 3D images; each of these isosurfaces corresponds to the local point spread function of the imaging system. The colour map indicates the maximum image intensity (normalised to 0 dB) for each of the 81 separate experiments. The blind spot indicated by the green dashed box is not reconstructed to avoid image artefacts due to overlap between signal and acoustical cross-talk. **Right column:** Compound image displaying the local point spread function obtained from 81 separate imaging simulations for an infinitesimally small point scatterer.

Both simulations and experiments indicate that in all three directions, the highest resolution was obtained on-axis (*x* = *y* = 0 mm), and that the resolution in all three directions decreased with increasing distance from this axis. This is visualised for a point target depth of *z* = 0.9 mm and a source pattern comprising concentric circles in Fig. 4. Within the interval $(|x|\le 2$ mm, $|y|\le 2$ mm), the experimentally obtained resolutions in the *x*, *y* and *z* directions varied between 97 to 343 μm, 179 to 286 μm, and 110 μm to 1.11 mm, respectively. Results obtained for all four source pattern geometries and point targets located at both *z* = 0.9 mm and *z* = 2.4 mm are given in Supplementary Fig. 3 and are summarised in Table 1; in all cases the highest resolution was obtained on-axis, and the *z*-extent of the point spread function and its spatial variation were reduced at greater depth.

**Table 1:** Minimum and maximum spatial extents (μm) of the 3D point spread functions obtained from 81 measurements along two planes located at axial depths of *z* = 0.9 mm and *z* = 2.4 mm.

**Source pattern**	*z* = 0.9 mm			*z* = 2.4 mm		
	*x*	*y*	*z*	*x*	*y*	*z*
Single circle	110→373	190→286	170→1159	152→426	177→342	122→505
Concentric circles	97→343	179→286	110→1110	157→350	167→357	88→475
Rectangular grid	100→357	176→302	110→1207	157→350	172→373	88→529
Randomised	102→365	197→316	115→1207	156→350	175→390	99→495

Point spread functions were measured for four different source location patterns, and their spatial extents are given as minimum value →  maximum value.

For all source patterns and point target depths, a slight discrepancy between simulations and experiments was observed in the image intensities recovered for each point target location. In simulations, the image intensity varied by less than 7 dB across the interval $(|x|\le 2$ mm, $|y|\le 2$ mm), whereas in the experimental results a larger variation of up to 13 dB was observed, in addition to a trend where the image intensity increased with increasing *y*. This trend was caused by the finite dimensions and slight misalignment of the pushpin: for pushpin positions with positive *y*-coordinates, the specular reflection off the oblique edge was recorded by the receiver and hence the phantom no longer appears point-like.

### 3D imaging

**Figure 5:**
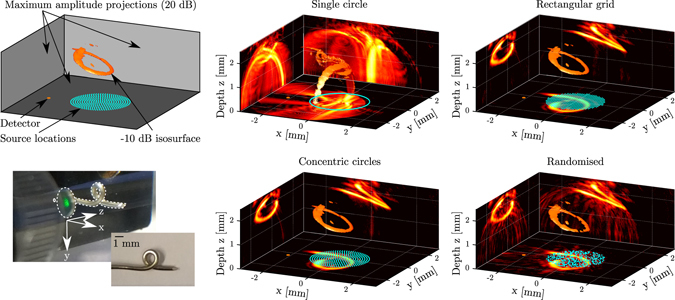
Three-dimensional (3D) images obtained with the all-optical pulse-echo imaging system using four different excitation patterns. A twisted metal wire (bottom left) was used as phantom. A similar visualisation and the same excitation patterns as in Fig. 3 were used; source locations are indicated in light blue, the receiver in orange, and the three panels show maximum amplitude projections on a logarithmic scale with a 20 dB dynamic range. The 3D volumetric renders show the −10 dB isosurfaces.

Images obtained of a looped wire phantom (the centre of which was placed at a depth of *z* = 1.8 mm) confirmed the high image resolution predicted by the point spread function measurements, and demonstrated the effect of the ultrasound transmission pattern on the image quality. When an ultrasound transmission pattern comprising a single circle was used, image artefacts dominated the image and consequently the phantom geometry could hardly be distinguished (Fig. 5). However, when either of the three source location patterns spanning the entire fibre bundle lumen was used, these artefacts were strongly suppressed and the phantom geometry was clearly recovered. Of the three source location patterns covering the fibre bundle lumen, the randomised pattern yielded the highest artefact levels and lowest image contrast. Higher image contrasts were obtained for patterns comprising concentric circles or a rectangular grid; both patterns resulted in a similar image quality. The same conclusions could be drawn when the phantom was placed at a depth of *z* = 3.3 mm (Supplementary Fig. 4); however, at this depth the phantom geometry was only partially recovered. The 3D nature of the phantom is best visualised in the animated representation available online in Supplementary Movie 1.

**Figure 6:**
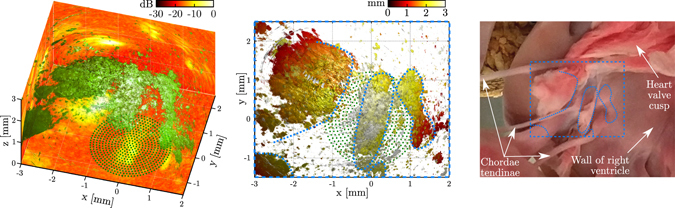
Three-dimensional (3D) image of *ex vivo* porcine cardiac tissue obtained with the all-optical pulse-echo imaging system. A photograph of the sample, containing myocardium, tendinae chordae (“heart strings”) and a heart valve cusp, is shown on the right. The left panel shows the −15 dB isosurface (green), and the three planes (*z *= 0 mm, *x *= −3 mm and *y* = 2.5 mm) display the maximum intensity projections (logarithmic scale) along the *z*, *y* and *x* directions, respectively. To improve visual depth perception, the middle panel displays the same −15 dB isosurface with the axial depth indicated in colour. The approximate locations of anatomical features are indicated in blue dotted curves (middle and right), whereas the source locations are indicated by green dots.

The volumetric image of porcine cardiac tissue (Fig. 6) yielded a field of view of 5 mm × 4 mm × 3 mm in the *x*, *y* and *z* directions, respectively. The maximum intensity projections revealed a contrast-to-noise ratio of 15 dB and a depth-dependent signal-to-noise ratio of up to 30 dB. While the surface of the anatomical features located within the field of view were accurately reconstructed, the low contrast-to-noise ratio resulted in a limited penetration depth of below 0.5 mm.

## Discussion

To the authors’ knowledge, this study presents the first demonstration of three-dimensional all-optical ultrasound imaging using a stationary, real aperture. By scanning focussed light across the proximal end of a semi-flexible coherent fibre bundle, ultrasound was generated photoacoustically in the corresponding region at the distal end. The resulting scanning configuration facilitated the implementation of arbitrary ultrasound array geometries using the same physical acoustical sources and detector, thereby enabling a direct comparison of the achieved image quality and artefacts. The absence of electrical connections greatly facilitated the fabrication of the miniature transducer array, and the use of fibre-optic technology renders the probe insensitive to electromagnetic interference. Together with an outer diameter of 3.5 mm, these advantages render the probe ideally suitable to endoscopic and laparoscopic applications, which are often performed with concurrent alternative image modalities such as MRI or CT.

The all-optical 3D ultrasound imaging system yielded high resolutions for four different source location patterns. However, the patterns studied in this work had inter-element spacings comparable to the acoustic wavelength, and hence violated Nyquist’s sampling criterium. Consequently, the geometry of the source pattern was found to have a profound effect on the image artefact levels, which suggests that the performance of the imaging system could be further optimised by reconfiguring the source pattern. For instance, patterns based on Fermat spirals^36^ or co-prime geometries^37^ might yield tighter focussing and lower artefact levels using fewer source locations, and could result in improved contrast-to-noise ratios and consequently greater penetration depths. In addition, aperiodic arrays could be implemented to suppress side- and grating lobes, as has previously been demonstrated at low frequencies (<2 MHz) using mechanically separated piezoelectric transducer elements^38–42^, and studied in simulation for 2D arrays^43^. The latter study envisioned a fully populated rectangular grid of CMUT transducers, and the inherent periodicity resulted in strong side-lobes and a high transducer complexity. At higher frequencies, such aperiodic arrays could be manufactured using a kerfless approach, where electrode patterning rather than mechanical separation is used to isolate elements^44, 45^. However, stronger mechanical cross-talk between neighbouring elements is typically observed for kerfless arrays than for conventional arrays^46^, resulting in a reduced image quality. Due to strong acoustical attenuation within the optically absorbing coating^47^ and the large impedance mismatch between the coating and the glass fibre bundle substrate, it can be expected that such mechanical cross-talk does not occur to a significant extent in the all-optical setup presented in this work; the surface pressure distribution is hence solely determined by the spatial confinement of the optical excitation.

Neglecting the effects of acoustical attenuation, the spatial resolution reported here is determined solely by the acoustical bandwidth and source aperture, and as such is independent of the location of the optical receiver. However, to maximise the signal-to-noise ratio, the detector should be placed close to the source aperture to minimise geometrical attenuation. Furthermore, the spatial resolution and penetration depth could be improved by employing image reconstruction algorithms that further exploit spatial coherence between the A-scans, such as short-lag spatial coherence (SLSC) imaging^48^ or the delay, multiply and sum (DMAS) algorithm^49^. Through changes in the optics, the size of the acoustical sources could be varied to further optimise the image quality. For instance, a lens with a shorter focal length could focus the excitation light more tightly, and hence allow for smaller acoustic sources. Ultimately, light could be coupled into individual fibres of the bundle, resulting in acoustic sources with a diameter of only 12 μm. This could generate an array comprising up to fifty thousand acoustic sources within a diameter of 2.70 mm, each with reduced directionality and sufficiently small inter-element spacing to avoid grating lobes. However, to avoid optical damage to the ultrasound-generating coating, the pulse energy would have to be reduced, thereby reducing the acoustical signal-to-noise ratio.

In this work carbon black spray paint was used as sound generating medium, as this simplified the deposition of a confluent and flat coating at the distal end of the fibre bundle. While the acoustic pressure and bandwidth generated by this coating resulted in high-resolution images, the performance of the spray painted coating strongly deteriorated after prolonged submersion due to water penetration (up to a ten-fold reduction in pressure after 22 hours, cf. Supplementary Fig. 1). Alternatively, composite coatings comprising polydimethylsiloxane (PDMS) and multi-walled carbon nanotubes (MWCNTs) exhibit 3.3 to 5.8 times higher optical-to-acoustic transduction efficiencies^20^ and are significantly more robust: they have been used by the authors with similar fluences and pulse repetition rates for weeks without a noticeable decrease in performance (data not shown). However, attempts at depositing such composite coatings on the distal end of the fibre bundle resulted in domed surfaces, and the resulting acoustic lensing deteriorated the image quality.

The multiple-source, single-receiver approach of the current imaging system has several limitations that could be addressed in future studies. One limitation is the lack of flexibility of the fibre bundle. This could be addressed by using a leached imaging bundle, which would enable interventional applications where a high level of flexibility is crucial. A second limitation is the lack of video-rate imaging. Indeed, the data for each image presented here were acquired in just under 4 s. However, using a modest laser pulse repetition rate of 10 kHz would allow for a scan rate of 17 volumes per second. Reconstruction of each 3D image took approximately 35 s, but by pre-computing delay matrices and employing GPU technology, this reconstruction could be accelerated significantly^50^. In addition, the number acoustic source locations, and hence the acquisition time, could be reduced through optimisation of the source pattern geometry. Alternatively, the inverse approach, using a single source and multiple receivers, is feasible by pairing a single optical acoustic source fibre with a fibre bundle comprising a Fabry-Pérot cavity at its distal end^51^, which could be interrogated using multiple beams to reduce the acquisition time^52^. If multiple sources and receivers are used simultaneously, both the acquisition time and the signal-to-noise ratio of the images could be improved. However, this approach would introduce significant additional technological complexities, and would require additional equipment that can be both bulky and costly.

This study demonstrates how optical technology can be employed to fabricate ultrasound imaging probes that are sufficiently small for endoscopic use, and yield high-quality 3D images of both phantoms and *ex vivo* tissue without the need for synthetic aperture scanning. The transducer array can be optically reconfigured to optimise the performance of the system in terms of resolution, contrast and signal-to-noise ratio. The presented approach shows great promise for achieving real-time 2D or 3D imaging of biomedical tissue with a fully reconfigurable acoustic source array that can be tailored to specific applications. In addition, rather than delivering light through a fibre bundle, a free-space setup where light is delivered directly to a coated surface can be used to achieve an acoustical array of which both the geometry and dimensions can be reconfigured to tailor the system to various imaging scenarios.

## Electronic supplementary material

Supplementary figures 1–4

Volumetric render of the image obtained from a wire phantom
