# Supplementary material for: A reconfigurable all-optical ultrasound transducer array for 3D endoscopic imaging
Source: Sci Rep. 2017 Apr 26;7:1208. doi: 10.1038/s41598-017-01375-2 (PMC5430692; doi:10.1038/s41598-017-01375-2)
Supplement: Supplementary file 1 — Supplementary figures 1–4 [file 41598_2017_1375_MOESM1_ESM.pdf]

Supplementary data - A reconfigurable all-optical ultrasound transducer array for 3D endoscopic imaging

Erwin J. Alles[1,*], Nora Fook Sheung[1], Sacha Noimark[1,2], Edward Zhang[1], Paul C. Beard[1], and Adrien E. Desjardins[1]

[1]Department of Medical Physics and Biomedical Engineering, University College London, London WC1E 6BT, U.K.
[2]Materials Chemistry Research Centre, UCL Department of Chemistry, London WC1H 0AJ, U.K.
[*]E.Alles@UCL.ac.uk

ABSTRACT

This document contains the supplementary figures to main manuscript *"A reconfigurable all-optical ultrasound transducer array for 3D endoscopic imaging"*.

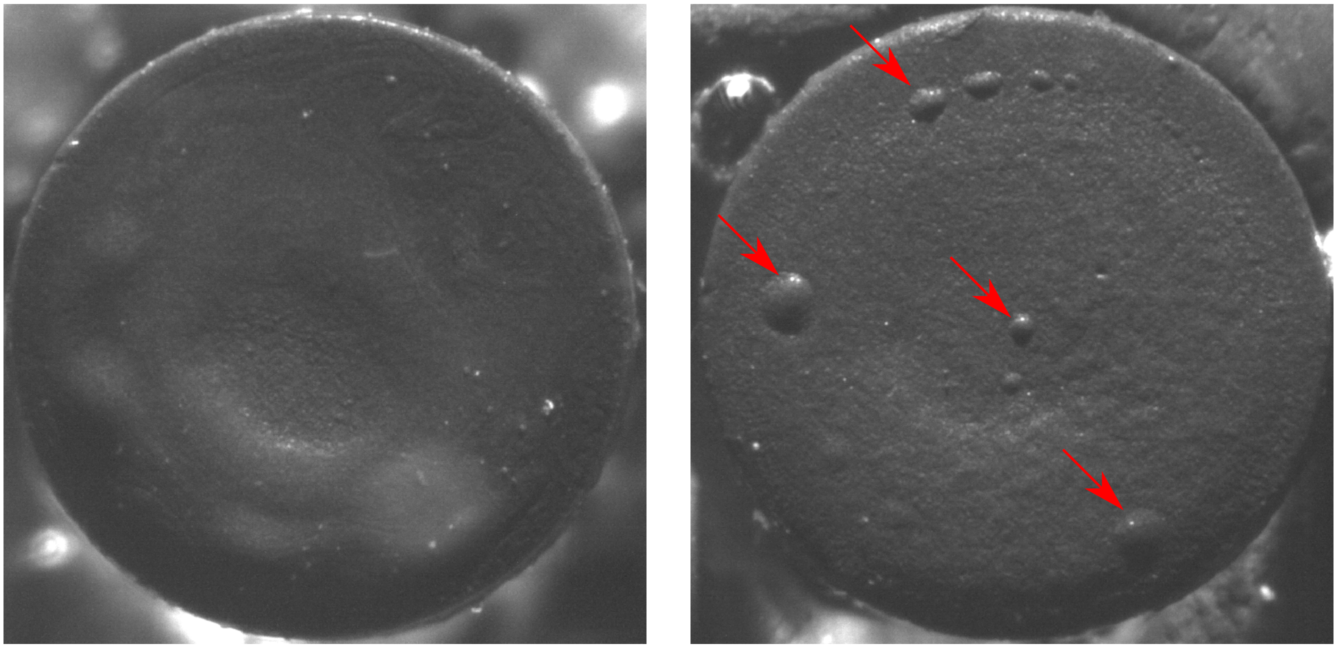

Supplementary Fig. 1. Optical microscopy of the coated distal end of the fibre bundle, acquired using grazing illumination. **Left:** Typical appearance of a deposited coating prior to submersion in water. Slight inhomogeneities of the coating thickness (within approximately 30 μm) across the surface can be observed. **Right:** After prolonged submersion (displayed case: 22 hours), water penetration caused the coating to locally "balloon out". The affected areas (red arrows) retained optical opacity, but generated ultrasound of a reduced amplitude (up to a factor of ten).

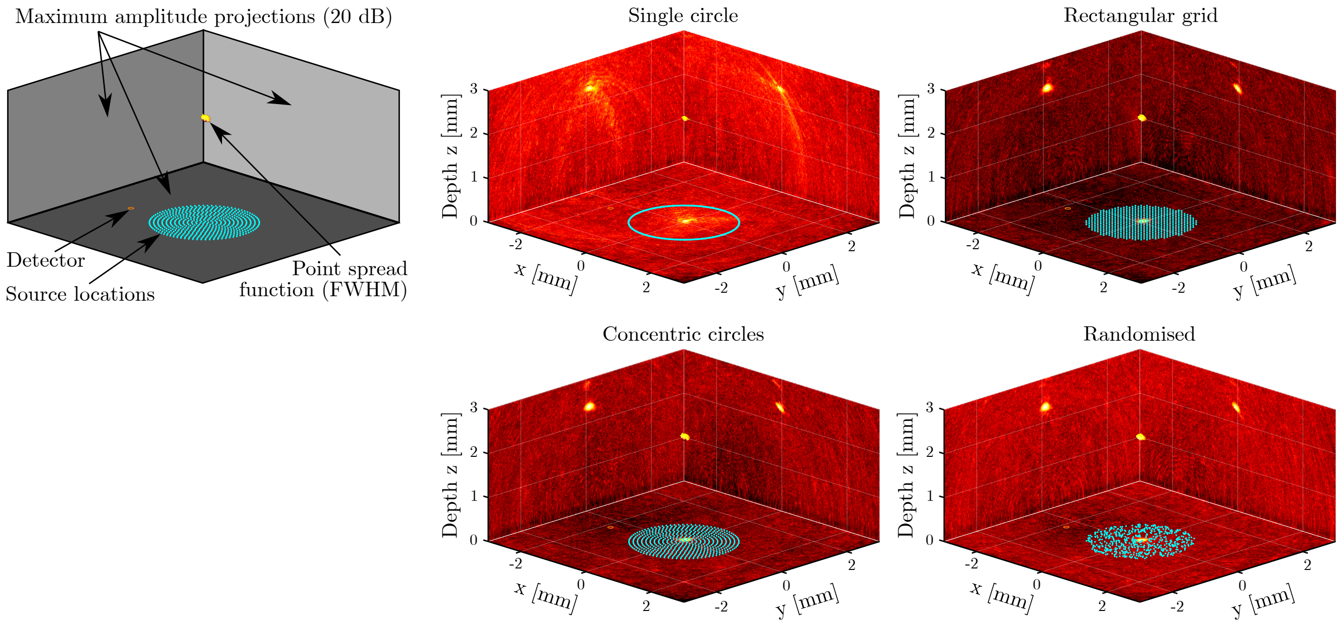

Supplementary Fig. 2. Images of the tip of a thumbnail obtained with the all-optical, three-dimensional pulse-echo ultrasound imaging system. The thumbnail tip was positioned at a depth of $z = 2.4$ mm, and four different source patterns were used to image this point target. The figure lay-out is identical to that of Fig. 3 of the manuscript.

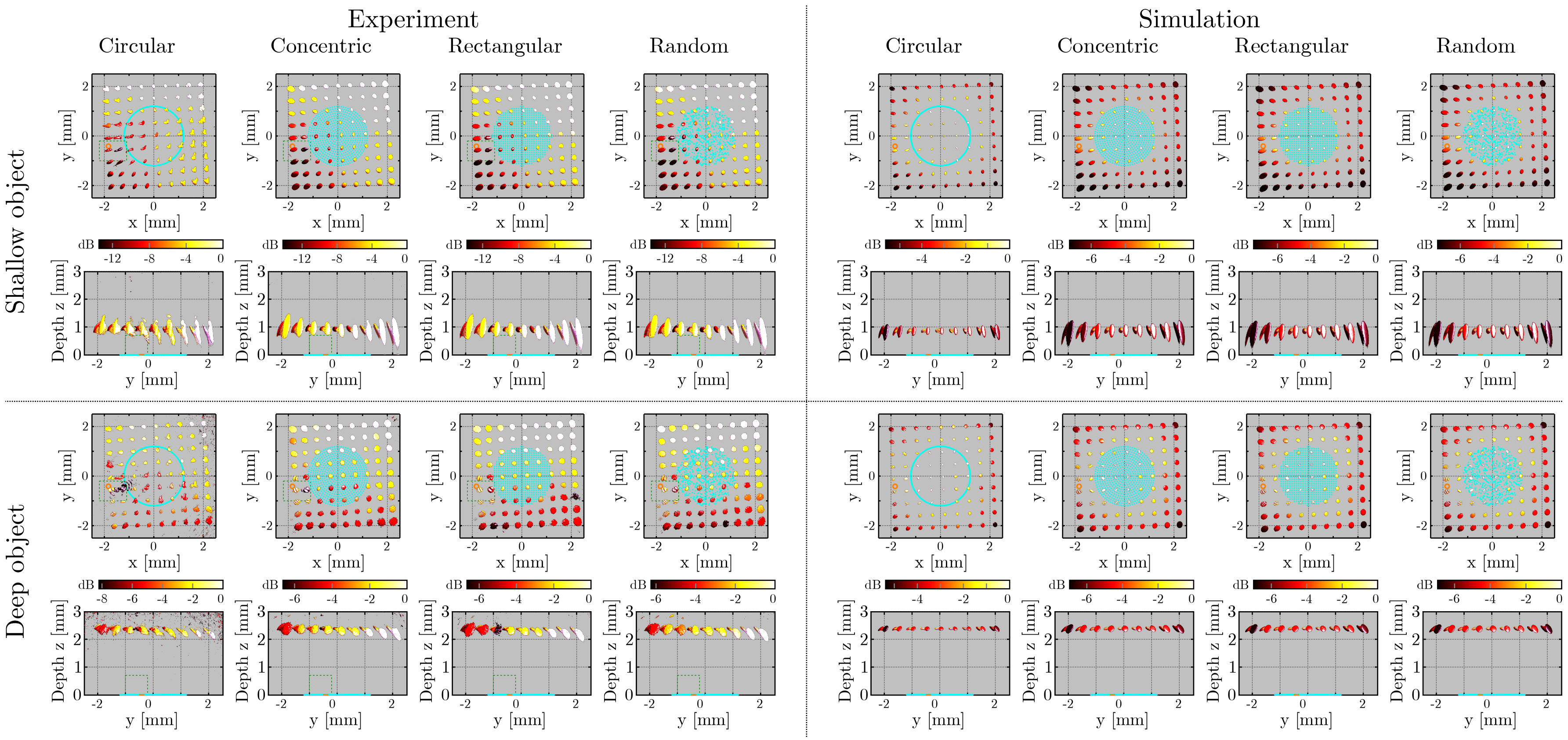

Supplementary Fig. 3. Measurements and simulations of the spatial variation of the point spread function, for four different source patterns and two different point scatter depths. The lay-out and visualisation of each panel are identical to those of the panels in Fig. 4 of the manuscript.

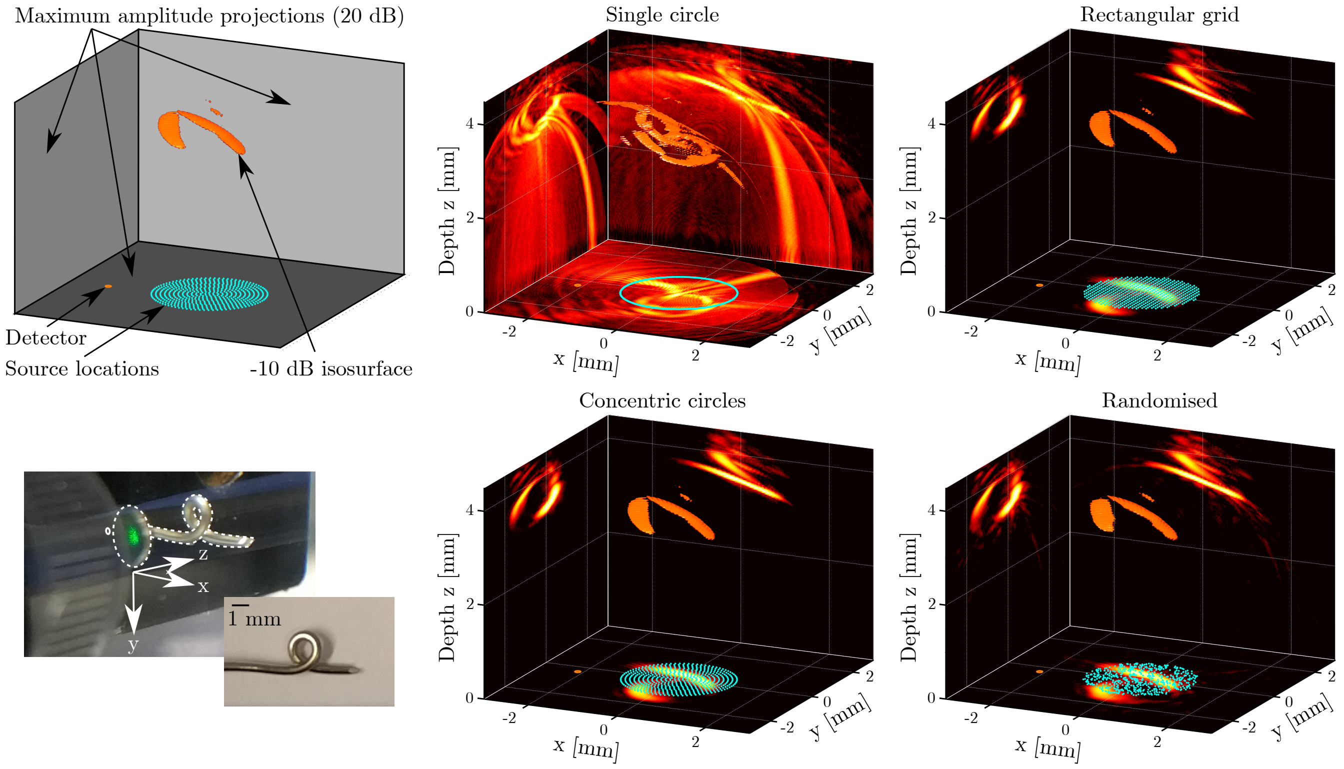

Supplementary Fig. 4. Three-dimensional images obtained using four different excitation patterns. A twisted metal wire (bottom left) placed at a depth of $z = 3.3$ mm was used as phantom. The lay-out and visualisation of the panels are identical to those in Fig. 5 of the manuscript.

Supplementary Movie 1. Animation of the 3D rendering of the image obtained from the wire loop phantom. The two panels display the same image rotated around different axes to facilitate interpretation. Source locations are indicated by red dots, the location of the detector is indicated in orange. Ticks along the axes represent millimeters.
